# Stabilization Effects of Natural Compounds and Polyhedral Oligomeric Silsesquioxane Nanoparticles on the Accelerated Degradation of Ethylene-Propylene-Diene Monomer

**DOI:** 10.3390/molecules26154390

**Published:** 2021-07-21

**Authors:** Traian Zaharescu, Ignazio Blanco

**Affiliations:** 1 INCDIE ICPE-CA, 313 Splaiul Unirii, 03138 Bucharest, Romania; 2 Department of Civil Engineering and Architecture, University of Catania, V.le A. Doria 6, 95125 Catania, Italy; iblanco@unict.it

**Keywords:** stabilization, EPDM, γ-irradiation, rosemary, POSS, antioxidant activity

## Abstract

In this work the analysis on the stabilization activities of some natural antioxidants (rosemary extract, capsaicin, quercetin or oleanolic acid) is presented. A similar contribution of an inorganic structure—polyhedral oligomeric silsesquioxane (POSS) nanoparticles—is also evaluated. The stabilization effects on the oxidation protection were investigated for several formulations based on ethylene-propylene-diene-terpolymer (EPDM). The samples were examined in pristine state or after γ-irradiation, when the accelerated degradation scission of polymer macromolecules followed by the mitigation of oxidation. Three evaluation procedures: chemiluminescence, FTIR spectroscopy and thermal analysis were applied for the characterization of stability efficiency. The delaying effect of oxidative aging in EPDM matrix is illustrated by the values of activation energy, which are correlated with the type and concentration of embedded compounds. The durability of studied EPDM formulations is discussed for the assessment of material life. The improved behavior of structured hybrids useful for the optimization application regimes is essentially based on the antioxidant properties of polyphenolic components in the cases of natural antioxidants or on the penetration of free radical intermediates into the free volumes of POSS.

## 1. Introduction

The capability of rosemary extracts and many other natural extracts to prevent material aging has been recognized for a long time. They are regularly used in the preparation of foodstuffs [1]. The composition that sustains the delay of oxidation by rosemary extract is already known [2]. The stabilization effects on reference material/paraffin [3], packaging polymers [4,5], medical materials [6,7], anticorrosive surface protection [8], inhibition of the oxidation in biodiesel [9] are already reported. These uses are based on the ability of active rosemary components or other natural extracts to block harmful processes fed by the diffusion of oxygen. The main component of antioxidant blends in rosemary extract—carnosic acid, a polyphenol [10,11]—has a large abundancy in different extracts. The summary of contributions brought about by the significant components of rosemary powders on the overall stabilization effect indicates these compounds as the efficient stimulants for the protection against oxidation [12]. The mechanism through which rosemary extract acts as an oxidation protector is already reported [3]. It must be emphasized that all derivatives of carnosic acid existing in the conversion chain of carnosic acid present similar antioxidant features with sufficient protection potential [3].

During the usage of polymers, as well as their long term storage under unproper conditions, these materials become degraded. The aleatory fragmentation of macromolecules generates reactive radicals. A low proportion react with each other, but the largest amount is oxidized by a self-catalytic process through peroxyl intermediates. The preservation of oxidation state at the lowest level requires a suitable compound capable of delaying the material ageing process. The protection interaction between free peroxyl radicals formed in the oxidation chain and antioxidant molecules is described by mobile protons being accompanied by the remote of peroxyl radicals of hydroperoxides. The molecules of various types of stabilizer compounds existing in rosemary extracts are reversibly converted into quinone and semiquinone configurations that continue the delay of oxidation as occurs in the case of carnosic acid. It was demonstrated that the activities of rosemary loadings are proportional to their contents [12]. The appropriate features related to the radical scavenging in degrading polymers are essential for the material durability. However, the efficiency is influenced by the rosemary oil harvesting period [13] and the type of procedure applied for the extraction of active fractions [2,14]. Similarly, polyphenols show their antioxidant potential by the protective activity of phenolic proton. Thus, their basic contribution guaranties satisfactorily the extension of material lifetime during inevitable thermal oxidation that occurs permanently. 

The sensitivity of a polymer to oxidation is mainly determined by the molecular configuration and the material’s microstructure (degree of crystallinity, state of entanglement) that condition the diffusion rate of oxygen. The addition of a proper filler can improve the oxidation resistance provided by the interference in the degradation mechanism [15]. During the oxidative ageing of polymers, various intermediate radicals are continuously generated. They are protected against oxidation by the scavenging activity of antioxidant molecules. This role is based on the availability of mobile protons of hydroxyl (phenolic) groups. It was demonstrated that a high efficiency limiting oxidative degradation is obtained when these protons are easily lost as happens in the case of rosemary extract [16]. The prevention of oxidation by rosemary extracts is controlled by the additive composition, the relative proportion of components in the added rosemary powder, the degradation conditions (temperature, weak sites on the polymer skeleton), the product history and the manufacturing technology used. The availability of rosemary extracts for breaking degradation chains can be ameliorated by the addition of other stabilizing components like polyhedral oligomeric silsesquioxane (POSS), whose protection mechanism is based on its cage effects for scavenging radicals.

In spite of the large attention paid to the effects induced by the presence of polyhedral oligomeric silsesquioxanes (POSSs), their stabilization activities were scarcely investigated. The influence of structured polymer materials [17,18,19], hybrid composition [20,21,22], preparation conditions [23,24] are the main directions that are envisaged by the majority of assays. Unfortunately, the contribution of POSS filler to the delay of oxidation in polymer materials was randomly reported [25,26]. An appropriate study on the antioxidant properties of POSS was previously published [27], where the stabilization efficiency of this inorganic structure was evaluated by the calculation of activation energy required for the protection of EPDM against accelerated degradation. However, the investigation of the proper contribution of POSS coupled with a classical antioxidant is not published yet. The promotion of this kind of compounds (inorganic filler accompanied by a hindered phenolic structure) in certain concerns offers pertinent solutions for several applications like packaging materials, medical wear, commodities and toys with suitable durabilities.

The investigation of protection effectiveness shown by various suitable compounds in ethylene-propylene-diene terpolymer (EPDM) provides generally applied information for about all polyolefins, a large category of engineering polymers. The thermal and radiochemical stabilities of EPDM are influenced by the molecular structure—the ratio between ethylene and propylene units [28]—as well as the nature and amount of diene that is the weakest site inside these configurations [29]. The presence of antioxidants for the improvement of service time of products is mandatory [30]. There is a large spectrum of antioxidants, either synthetic compounds [30,31] or natural extracts [15,32,33].

The accelerated degradation caused by the γ-irradiation is an adequate procedure for the evaluation of antioxidant properties of additives because they can interfere with the chain intermediates by breaking the self-catalytic oxidation process. The addition of antioxidants in the formulations of polymers allows one to extend their durability. The selection of the appropriate molecular structure of the stabilizer relative to the intensity and duration of the stressor action can be successfully achieved [34] by activity investigation. Accordingly, the commercial products gain extended lives if the screened degradation protection is effective and efficient.

## 2. Results

The protection content presents always a specific activity that preserves the oxidation state of a polymer material against its ageing over long time use. The selection of available alternatives in respect with the improvement of kinetic parameters of degradation allows one to foresee the extension of product durability. The large variety of compounds having antioxidant properties allows the selective investigation on different structures, which are beneficial for a large spectrum of applications with several advantages related to the evolution of functional targets.

In this study, the antioxidant activities of several organic structures (natural extracts) and an inorganic configuration (vinyl-polyhedral oligomeric silsesquioxane, named POSS later on) are analyzed and compared by means of chemiluminescence and thermal analysis. Therefore, reliable comments on the prevention of oxidative damage for packaging materials or on the addition of recycled polymers with favorable effects on the environmental protection are feasible. The information provided by these investigations consists of the nature and concentration of additives. The temperature range under which the degradation was achieved is also examined.

### 2.1. FTIR

**Figure 1:**
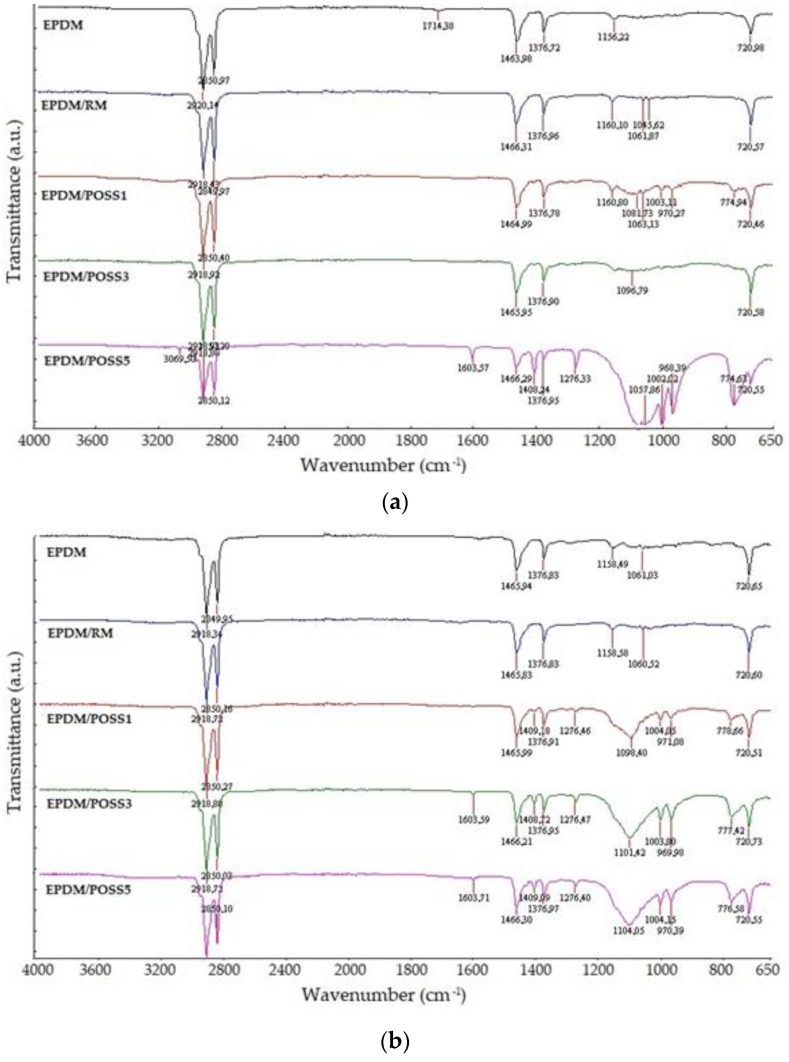
FTIR spectra of studied EPDM formulations. Irradiation doses: (**a**) 0 kGy and (**b**) 50 kGy.

A spectroscopic characterization of compositional analysis was first carried out to verify the presence of the molecular filler in the polymer. FT-IR measurements show unlike for the composites with different consistencies than for the spectra of neat EPDM and EPDM/RM (rosemary) (Figure 1a,b). The band at about 1100 cm^−^^1^ related to the asymmetric Si−O−Si stretching is associated with the POSS cage [35,36] confirming, at least qualitatively, the presence of POSS molecules in the EPDM composites. It confirms the involvement of inorganic structure simultaneously with organic additive in the slowing down the alteration of polymer substrate.

It is worth noting that the band associated with the presence of POSS molecules is clearer for the composites irradiated at 50 kGy (Figure 1b), whilst for the non-irradiated composites the greater or lesser presence of POSS molecules seems to influence the intensity of the peak (Figure 1a), which increases passing from the composite at 1% of POSS to that at 5% of POSS.

### 2.2. Thermal Behaviour

The calorimetric investigation was carried out in a differential scanning calorimetry (DSC) from room temperature up to 150 °C. It shows no significant changes in the melting behavior of the composites with respect to the virgin polymer, which was at about 35 °C for all the investigated samples (Table 1) in agreement with literature reports [37].

**Figure 2:**
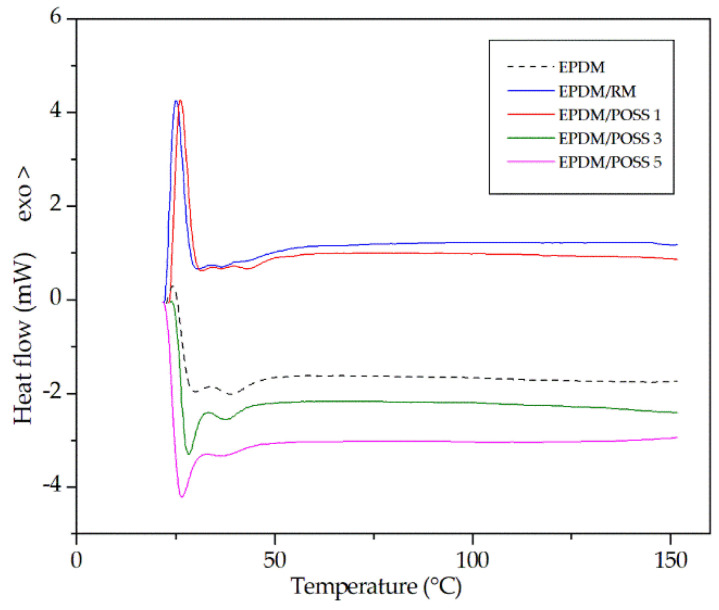
Differential scanning calorimetry curves for irradiated EPDM and prepared composites.

In the case of DSC measurements a slight increase in the melting temperature may be observed for the irradiated samples, both for pristine EPDM and for its composites. This behaviour is in agreement with literature evidences [38,39], which showed that the melting points of γ-irradiated samples were improved by the radiation induced crosslinks leading to an enhancement of crystallinity [40,41]. Contrariwise, the quantity of molecular filler does not seem to affect the melting behaviour, that this consideration is quite similar for the EPDM based composites at different POSS content. For the sake of brevity DSC profiles of the irradiated samples are reported in Figure 2.

The thermal characterization was completed by degrading EPDM and EPDM composites in a thermogravimetric (TG) system from room temperature up to 700 °C. TG analysis was carried out aiming at evaluating the thermo-oxidative behaviour of the control EDPM and its composites. To this purpose the temperature at 5% mass loss (T_5%_) and the residue percentage at the end of the thermogravimetric analysis (TGA) must be considered as main parameters to evaluate the thermal performance of the investigated compounds. In particular, T_5%_ was preferred to the most known initial decomposition temperature (T_i_), because this latter is strongly affected by the shape of the TG descending mass loss curve whilst T_5%_ represents, in our experience [42,43], a fair value of mass loss whereby a material can begin to be considered degraded.

**Figure 3:**
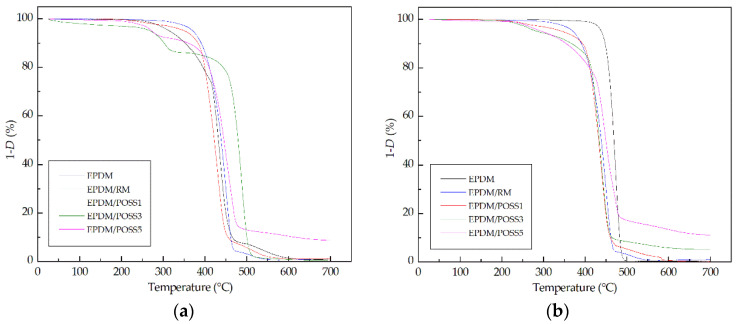
Thermogravimetric curves for the EPDM formulations. (**a**) unirradiated samples; (**b**) the samples subjected to a γ-dose of 50 kGy.

In the considered temperature range (25–700 °C) the degradation mechanism is completely different for the neat EPDM and the composite with rosemary (RM) with respect the POSS composites (Figure 3).

**Figure 4:**
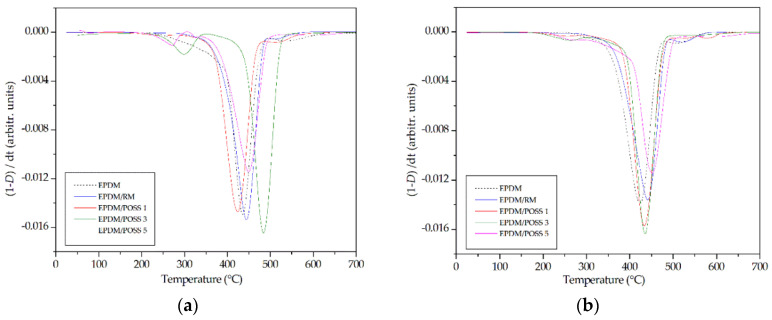
Derivative Thermogravimetric curves for the EPDM formulations. (**a**) unirradiated samples; (**b**) the samples subjected to a γ-dose of 50 kGy.

Derivative TG thermograms show that EPDM and EPDM/RM samples degraded in a single stage, whilst EPDM/POSS composites showed two different stages of degradation (Figure 4).

Analyzing the thermal paremeters reported in Table 1, namely the temperature at 5 phr mass loss and the residue at 700 °C a couple of considerations can be put forth:

- The presence of rosemary and POSS at 1% enhances the resistance to the thermal degradation, as testified by the increase in T_5%_ values, which was more pronounced for the non-irradiated samples (about 60 and 40 °C, respectively) than for irradiated ones (10 and 7 °C, respectively).
- On increasing the POSS amount in the composites a dramatic decrease in T_5%_ was observed for the composites with POSS at 3 and 5 phr respectively, probably due to aggregation phenomena that reduce the ability to be dispersed in the matrix of POSS molecules.
- the EPDM composites with 3 and 5 phr of POSS presented at the end of TGA a solid residue at 700°C being increased as a function of the filler amount in the hybrid composite. FTIR analysis was carried out on the solid residue showing the typical band associated with the presence of silica [44,45]. The presence of this residue at the end of the experiment confirmed the poor interaction with the matrix as POSS content increases.

### 2.3. Chemiluminescence Investigation

#### Pristine EPDM Formulations

The chemical composition of materials plays an important role in the progress of oxidation due to the controllable reduction of oxidation rates. The unmodified polymer present performances that are unsatisfactory for a long life or for the operation under unfriendly conditions. The advance in the oxidation state is described by the shape of isothermal CL spectra, which allow the identifications of occurred modifications in the degradation behavior of polymer. The thermal regime is dictated by the performances of additive whose sensitivity on the restriction upon oxidation limits the evolution of inhibition which is illustrated by the specific values of kinetic parameters.

**Figure 5:**
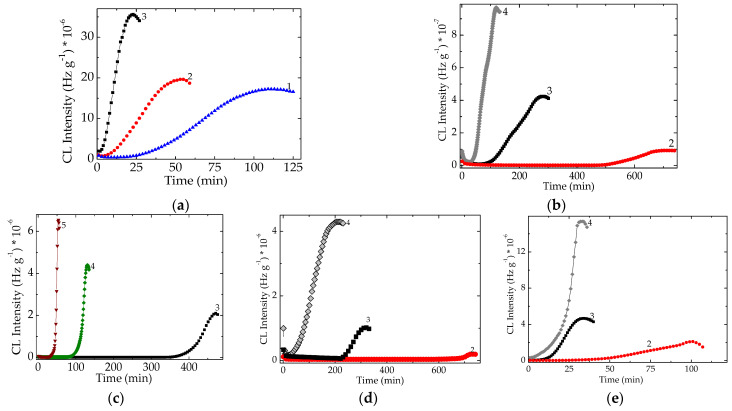

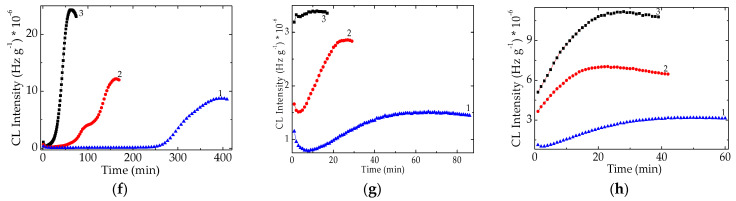
Isothermal CL spectra obtained on various compositions of EPDM samples. (**a**) pristine EPDM; (**b**) EPDM/rosemary; (**c**) EPDM/capsaicin; (**d**) EPDM/quercetin (**e**) EPDM/oleanolic acid; (**f**) EPDM/POSS 1 phr; (**g**) EPDM/POSS 3 phr; (**h**) EPDM/POSS 5 phr. Irradiation dose: 0 kGy. Testing temperature:(**1**) 160 °C; (**2**) 170 °C; (**3**) 180 °C; (**4**) 190 °C; (**5**) 200 °C.

The satisfactory prediction in the evaluation of protection efficiency can integrate the studied additives into the class of efficient antioxidants. The large differences that exist between the levels of oxidation delays at high temperatures (Figure 5) in respect with the pristine EPDM may be remarked due to the protection activities. The appropriate protection effects are obtained with inorganic compound POSS at consistent loading (1 phr), with natural extracts—rosemary, capsaicin, quercetin or oleanolic acid. They are good candidates for the preservation of polymer material integrity during its handling under harmful conditions. These results are somewhat expected, because these additives present commendatory behaviors for sustainable inhibition potential.

As it can be noticed from Figure 5, evident antioxidant activities are obtained at lower testing temperatures. While the polyphenolic structures work efficiently at the temperature higher than 170 °C, POSS acts adequately at some lower temperatures. The greater POSS contents, i.e., 3 and 5 phr, are the formulations that promote rather oxidation that is shown by all isothermal spectra unproper for screening oxidation. In contrast with pristine polymer, the improved formulations keep constant the lowest oxidation states on longer periods, the more extended oxidation induction times.

The analysis of stabilization effectiveness of natural extracts emphasizes the dominant influence of phenolic positions. The possibility of rosemary active component to be converted into other antioxidant structures increases the protection periods. Nevertheless, the chemiluminescence emissions are higher with an order of magnitude in respect with the values for the other tested natural compounds.

The ability of the molecules from natural compounds to operate as suitable antioxidants is associated with the facile substitution of protons from their phenolic moieties [46]. The protection activity of POSS is basically sustained by its possibility to scavenge oxidizing free radicals inside the spatial configuration [47]. It is obvious that the first mechanism is easier to achieve, while the admission of voluminous fragment from scission of polymer macromolecules limits the stabilized fraction. The examination of the CL isothermal spectra recorded on improved EPDM points out the necessity of minimal concentration of foreseen additives by which the afforded degree of adjustment can be obtained. Indeed, the origin of restrained stimulation may be found in the rate and strength of scavenging, by which the oxidizing intermediates are blocked on the seeming contact positions. The seems to assume that boundary conditions related to the molecular structure exist for rendering the reactive radicals onto stabilized forms.

Starting from the great values of temperatures at certain investigated samples, it may be foreseen that the studied additives have a worthy consequence on the proper extension of polymer durability at low temperatures, especially at room temperature. Like other polyphenolic structures, i.e., compounds belonging to a large variety of natural extracts (di- and triterpenes, simple phenolics, phenolic acids, flavonoids [48,49]), the studied molecular structures satisfy the sharp condition of good protectors against degradation. The mitigation of oxidative alteration is always solved by chain breaking that is effectively achieved by intrinsic interactions between the blocking structures and the potentially oxidized intermediates. The structural similarity between various antioxidants like our tested compounds do not define their capacity of protection. The correlation between the incorporating stabilizers and their antioxidant activities is already pointed out [12]. Therefore, the selection of natural extracts containing relevant active components is grounded on two structural groups: *ortho*-hydroxy configuration and the conjugation of double bonds with a 4-oxo function. They allow the preservation of thermal stability of these antioxidants at high temperature like our experimental conditions simultaneously with the remoting free radicals from the degradation chain.

The capability of any antioxidant based on the delaying, retarding or preventing oxidative degradation is naturally reported in respect with pristine host material. Figure 5 illustrates the individual contributions of various kinds of available structures on the effective protection of polymer, EPDM, as the most proper, convenient and cheap method for the improvement of long-term stability of new, used and recycled material, based on their friendly compatibility with environment and human body.

The durability improvement with these EPDM compositions may be easy extended to the other polymers by the present solution, which are degraded by radical mechanism like all polyolefins. The present results are useful for the most of commercial compositions that are manufactured with polyethylene and polypropylene. Their degradation chains are efficiently broken by the oxidation intermediates whose decay is accomplished by the suitable antioxidants like these present structures.

**Figure 6:**
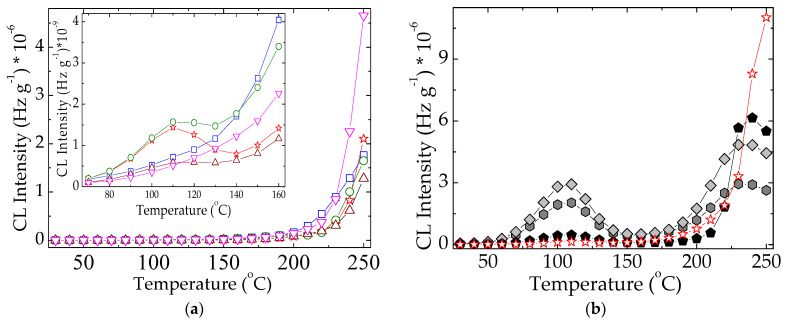
Non-isothermal CL spectra belonging to various compositions of EPDM samples. (**a**) red star: EPDM; blue square: EPDM/rosemary; olive cercle: EPDM/POSS 1 phr; wine triangle: EPDM/POSS 3 phr; magenta reverse triangle: EPDM/POSS 5 phr. (**b**) red star: EPDM; light grey rhomb: EPDM/capsaicin; dark grey hexagon: EPDM/quercetin; black pentagon: EPDM/oleanolic acid. Irradiation dose: 0 kGy. Heating rate: 10 °C min^−1^.

The modification in the oxidation temperature of evaluated hybrids has the stability consequence that makes difference between the protection activities of studied compounds. At 110 °C a peak appears demonstrating that the hydroperoxides generated by the oxidation of free radicals at lower temperatures are decomposed. However, the oxidation goes naturally on and the accumulation of these intermediates leads at the increasing CL emission at the temperatures exceeding 220 °C. In spite of the presence a similar maximum in the case of EPDM/POSS 1 phr, this formulation does not act as an oxidation inhibitor even though this compound brings about a significant delay of degradation under other circumstances [27]. An opposite behavior is shown by the sample containing POSS 3 phr, which has a satisfactory contribution at this heating rate on the propagation of degradation (Figure 6a). On the other hand, the stabilization effect of natural extract (Figure 6b) becomes effective by the participation of mobile protons from phenolic structures. Indeed, the mechanistic difference between the action of natural extracts and inorganic POSS consists of the dependence of radical scavenging by the volume hindrance in the vicinity of antioxidant reactive boundary. Despite good results in the delaying of degradation, all the inspected effects correlate the inactivation of formed free radicals in respect with diffused oxygen by various manners of ageing prevention. The degree of hindering oxidation is obviously obtained as the result of caching strength by which the generated fragments are obstructed to interact with oxygen. In spite of the major dissimilarities between the two different categories of antioxidant structures, the overall evaluation of stabilization effects provides similar results on a large temperature range, from room temperature up to 200 °C.

There are essential differences between the evolution of oxidation for inorganic and polyphenolic antioxidants (Figure 6). While a prominent maximum appears at 110 °C in the CL non-isothermal spectra, shoulders with various shapes illustrate the formation of hydroperoxides in unlike amounts. The other discrepancy between the two classes of stabilizers consists of the decay of hydroperoxides at higher temperatures. If in the cases of polyphenols (Figure 6b) their decomposition exceeds the rate of formation up to 175 °C, the competition between the formation and the decay of these intermediates affects the further evolution of degradation (Figure 6a).

Another dissimilarity between the two types of protectors, whose stabilization mechanisms occur not basically assorted, appears at high temperatures exceeding 200 °C. While the organic structures of polyphenols present an CL intensity peak associated with an advanced degradation, the continuous smooth increase in the CL emission caused by the decomposition of hydroperoxides becomes visible. The evolution of local concentration of degradation intermediates and the more efficient antioxidant activities of natural extracts are accordingly correlated.

**Figure 7:**
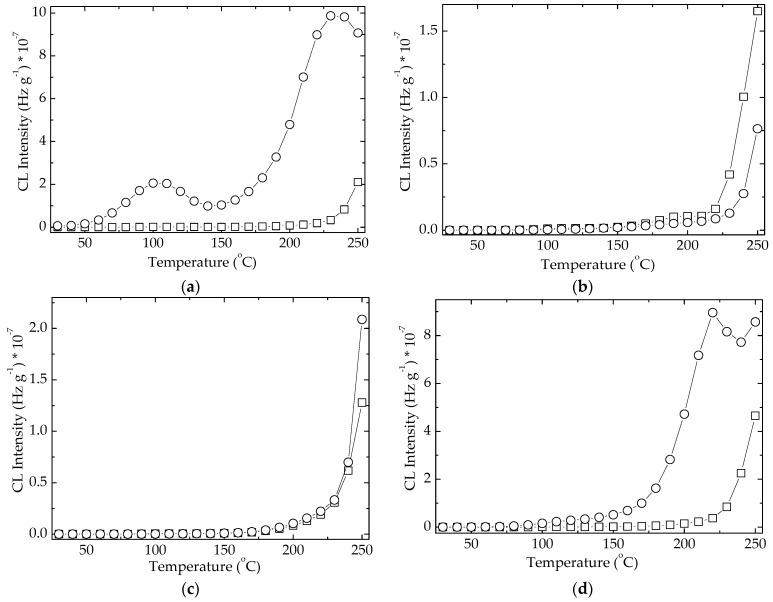

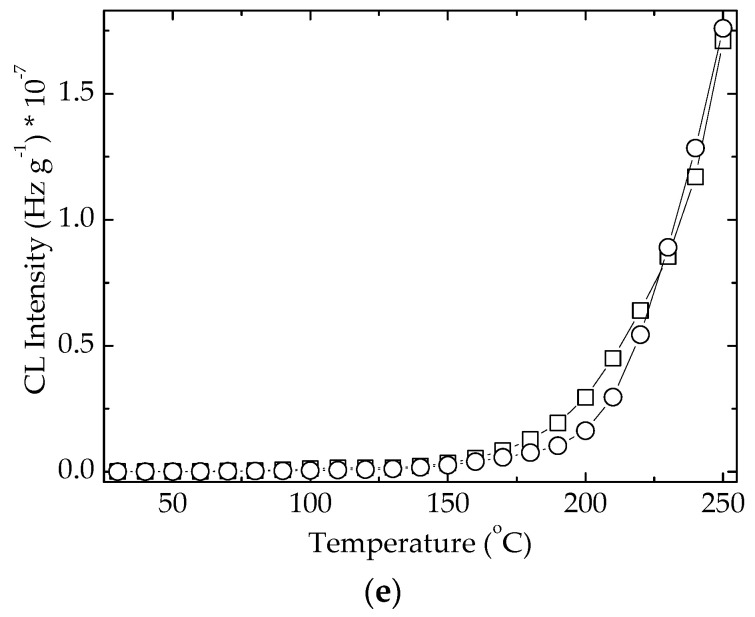
The comparison of nonisothermal CL spectra belonging to various compositions of EPDM samples. (**a**) neat EPDM; (**b**) EPDM/POSS 1 phr; (**c**) EPDM/POSS 3 phr; (**d**) EPDM/POSS 5 phr; (**e**) EPDM/RM (square) dose 0 kGy; (circle) dose 50 kGy. Heating rate: 10 °C min^−1^.

The comparison of non-isothermal CL spectra at two important doses (0 kGy for normal applications and 50 kGy for severe operation conditions) reveals the contribution of additives on the protection of EPDM substrate. If the advanced degradation occurs in neat EPDM, the presence of hydroperoxides is indicated by the shoulder at 105 °C (Figure 7a). It does not appear in the other similar spectra (Figure 7) because of the stabilization activities of additives. This proof defines the antioxidant feature for all additives. However, some differences between the studied compositions exist. The low concentration of POSS (1 phr) brings about the most efficient protection against polymer oxidation over the whole temperature range (room temperature/250 °C), while rosemary shows a satisfactory antioxidant activity up to about 220 °C. The higher loadings with POSS diminish the protective contribution of filler on the polymer. In the case of the higher concentration of POSS (5 phr), the pro-oxidation activity is displayed even at moderate temperatures (80 °C).

The large discrepancies in the evolution of degradation for two compositions (neat formulation and modified EPDM with POSS/5 phr) indicate the lack of any delaying action by the basic polymer or component.

## 3. Discussion

The modifications induced by oxidation describe the structural changes by which the energetic stressors, for example heat or exposure to γ-rays, accelerate material ageing. Thermal properties or chemical compositions are discussed. The contribution of added compounds in the formulations of polymeric materials are essential for the evaluation of stability evolution. The obtained results highlight the worthy importance concerning the association of antioxidant structure with the stability adjustment of polymers.

The thermal features reflect the physical interaction between the basic material—polymer and the present additive or filler. If the unirradiated samples present a small variation in the melting temperatures, the radiation treatment produces similar values of T_m_ that indicates the existence of similar assignments of oxidation products. The increased temperature values at 5% mass loss state of the samples containing an efficient stabilizer (rosemary or POSS 1%) illustrate an appropriate effect on the inhibition of oxidation. If the similar values for the three samples containing different loadings of POSS are compared, the antioxidant effect may be clearly noticed for unirradiated compositions. The main consequence of antioxidant activity seems to be the prevention of degradation. The most illustrative values that depicts the lack of hindrance of degradation are residue percentages of hybrid material, EPDM/POSS 5 phr, whose figures are very high. This image may be explained by the ease of degradation without any contribution of the filler for the conservation of the oxidation state.

The stimulation of antioxidant activities is modulated by the energetic conditions under which the reactions are accomplished. The activation energies that are involved in the interaction between the active positions of antioxidant and the intermediates are protected against their reactions with diffusing oxygen are reliable measure of efficiency. In Table 2 the values of activation energies explain the level of the individual contribution for the initiation of improved stability. The stability investigations pointed out the potential of various structures for the delay of oxidative degradation. For the samples containing different loadings of POSS powder, the increase in the filler concentration diminishes the stabilization efficiency. This feature may be ascribed to the competition between various free radical intermediated for the feeling of available free volume inside the POSS configuration. This behavior is also correlated with the size of intermediates which limits the number of entities abstracted from the oxidation chains [50]. On the other hand, the removing proton antioxidants like the present polyphenols are versatile stabilizers for unproper degradation conditions. Because the local concentration of free radical intermediates is very high during the exposure of material to the action of high energy radiation like γ-rays, the extension of obtained antioxidant behaviors over the thermal and photo-degradation is quite appropriate.

The advantages brought about by the modification of polymer formulations are correlated with the high safety of handled products, especially perishable food. The high antioxidant activities offered by the presence of rosemary extract, quercetin, capsaicin or POSS 1 phr guarantee long term durabilities, which are suitable for the efficient protection of polymer as well as the preservation of oxidation states for the packaged products. The cumbersome task of stabilization is simplified by the intrinsic contribution of additives to the tightly blocked hydroperoxides. Their abundancy can shorten the material life times, but the inhibition of oxidation is practically obtained by the intervention of active sites on the abstraction of appearing radicals involved in the propagation of degradation.

The wide range of antioxidant structures presents different levels of activities, but these characteristics regarding the durability performances would be important in the same measure for engineering polymers or for biopolymers capable to protect perishable products like food or beverages.

## 4. Materials and Methods

### 4.1. Materials

**Figure 8:**
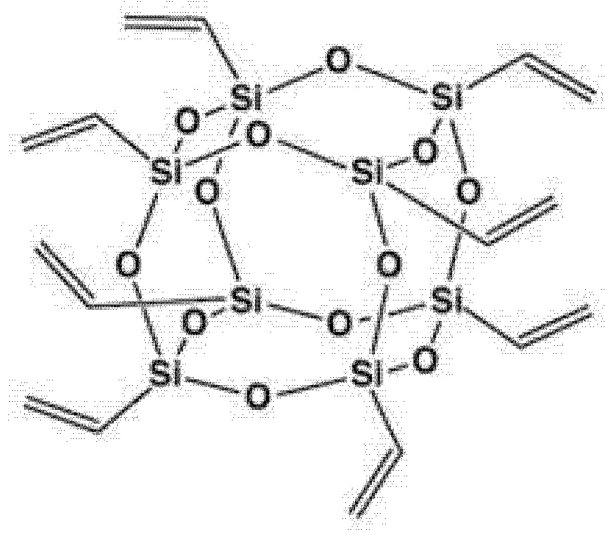
Spatial structure of (octavinyl—polyhedral oligomeric sisesquioxane) POSS.

Polymer substrate is ethylene-propylene-diene monomer (EPDM), produced by DSM Elastomers (Heerlen, The Netherlands) as KELRAN 8550. The molecular chains consist of two thirds of ethylene and one third of propylene, In the molecular structure a concentration of 5.5 wt% of diene (5-ethylidene-2-norborene) is incorporated. POSS (octavinyl-polyhedral oligomeric sisesquioxane) provided by Sigma Aldrich (Burlington, CA, USA) was used as an inorganic antioxidant, whose spatial structure is presented below (Figure 8).

**Figure 9:**
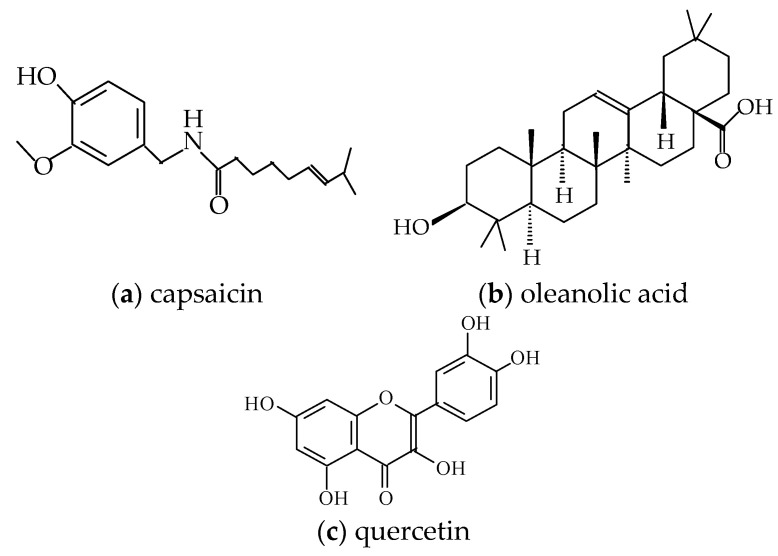
Molecular structures of (**a**) capsaicin, (**b**) oleanolic acid and (**c**) quercetin.

Rosemary extract was obtained in our laboratory by extraction from rosemary leaves. The powders of quercetin, capsaicin (≥95%, from *Capsicum* sp.) and oleanolic acid (≥97%) (see below their structures) were also provided by Sigma Aldrich (Figure 9).

### 4.2. Sample Preparaton and Irradiation

Rosemary leaves (about 10 g) were dried and placed in an Erlenmeyer flask together with ethanol, maintaining a ratio of rosemary and extracting solvent of 1:10 *w*/*v*. Maceration was carried out at room temperature for 120 h, by continuous shaking. The liquid was separated from the leaves by filtration and followed by evaporation under vacuum to remove the residue solvent, thus the obtained sample has been used for paraffin modification (0.25 wt%). Isothermal chemiluminescence determinations were performed in air at 160 °C by a Lumipol-3 instrument and the radioprotective capability of the rosemary extract was analyzed on a batch of 60 white mice. In agreement with the literature [51] the antioxidant activity of the rosemary extract should be due, mainly (over 90%), to some phenolic diterpenes such as carnosic acid, carnosol, rosmanol, isorosmanol, rosmadial, epirrosmanol, rosmaridiphenol, rosmariquinone. The pearls of EPDM were taken into chloroform solution for the removal of insoluble fraction from a clear polymer solution. The content of polymer was firstly determined for the calculation of the preparation of stabilized compositions. The appropriate loadings (1, 3 and 5 phr of POSS, 1 phr of rosemary) and 0.5 phr of quercetin, capsaicin and oleanolic acid) were added in separate fraction of mother EPDM solution. Indeed, each composition was separately prepared. The measuring specimens were obtained by the elimination of chloroform by volatilization at room temperature, when the thin layers were formed in the aluminum round pens for chemiluminescence investigation and 1 mm thick foils for thermal analysis. Due to the inactive feature of aluminum, surface during thermal measurements, this metal is preferred for the preparation of pans.

The accelerated degradation of samples was achieved by the γ-exposure in a irradiation room provided with a ^60^Co source, whose specific dose rate was 0.6 kGy h^−1^. The 50 kGy irradiation was decided because it covers enough dose for the sterilization application, also a sustained degradation for the evaluation of the strength on oxidation or any other hazardous conditions. The measurements were accomplished immediately after the end of radiation treatment for avoiding the uncontrollable alteration of results.

### 4.3. FTIR Analysis

Fourier transform infrared (FTIR) spectroscopy was carried out in a Spectrum 100 instrument (Perkin Elmer, Waltham, MA, USA). FTIR analysis was performed to check the presence of POSSs in the prepared samples, at room temperature, from 4000 to 650 cm^−1^ with a resolution of 4.0 cm^−1^. A universal ATR sampling accessory was used for measurements, which were made directly on the samples without further treatment.

### 4.4. Differential Scanning Calorimetry (DSC)

A Shimadzu DSC-60 (Shimadzu, Kyoto, Japan) calorimeter was used to evaluate the melting of the samples, after the calibration in temperature and enthalpy using indium, tin and zinc standard materials. The sample was placed in punched aluminum crucibles, and subjected to a heating rate of 10 °C∙min^−1^ from room temperature up to 150 °C in static air atmosphere. About 5 × 10^−3^ g of sample was examined each time, the DSC scanning was repeated three times for each sample and the averaged values was considered, the maximum difference between the average and the experimental values being within ±0.5 °C.

### 4.5. Thermogravimetric Analysis (TGA)

The thermal profile of EPDM and its composites was evaluated with a TGA 1 Star System (Mettler Instrument, Greifensee, Swizterland), after performing calibration using the change of the magnetic properties of three metal samples (Isatherm, nickel-alloy and Trafoperm 86) at their Curie points (148, 355 and 750°C, respectively). To avoid the error in the mass determination due to the reduction of the buoyancy force on increasing temperature a blank scan with an empty pan was carried out before the measure. These data were then subtracted from the sample scans, so obtaining corrected degradation thermogravimetric (TG) curves. For each determination about 5 × 10^−3^ g of sample was put into an open alumina crucible and heated from room temperature up to 700 °C, by using a scanning rate of 10 °C∙min^−1^. The degradations were carried out in flowing air (0.06 L∙min^−1^) atmosphere. TG scans were performed in triplicate, and the considered values were averaged from those of three runs, the maximum difference between the average and the experimental values being within ±0.5 °C. Our TG data were plotted as percentage of undegraded sample, (1–*D*) % against temperature, where *D* = (Wo − W)/Wo, and Wo and W were the masses at the starting point and during scanning.

### 4.6. Chemiluminescence (CL)

The stability determinations by chemiluminescence run in a specialized LUMIPOL 3 spectrometer (Slovak Academy of Science, Bratislava, Czech Republic). Non-isothermal measurements were performed at a convenient heating rate (10 °C min^−1^) and isothermal determination on the temperature range between 160 °C and 200 °C according with the stabilization efficiency. Aluminum pans are suitable for CL because they do not influence the oxidation state of polymer sample during measurements and this metal does not emit any radiation which would be spoil the results. The CL results are reliable due the proportionality between the number of emitted and the amounts of oxidizing intermediates [52,53]. The activation energy values were obtained by means of known Arrhenius relationship using the oxidation induction times (OIT) as basic kinetic parameter. 

## 5. Conclusions

This study analyzes the contribution of various compounds with antioxidant properties on the oxidation delay of ethylene-propylene-diene monomer, as a representative example of packaging materials. The present results depict the protection efficiency by means chemiluminescence procedure by which the activation energies of oxidation delay were calculated. This important kinetic parameter orders the capacity of various molecular structures for the prevention of oxidative degradation in the following sequence:

rosemary (1 phr) > POSS (1 phr) > quercetin (0.5 phr) > capsaicin (0.5 phr) >

> POSS (3 phr) > oleanolic acid (0.5 phr)

The actual antioxidant activities of studied systems do not intrinsically depend on molecular structures i.e., the protection mechanism. The preservation of oxidation level at the initial state is consistent with the molecular capacity of scavenging free radicals resulting from the scission of polymer macromolecules. It must be emphasized that neither high experimental temperatures nor γ-radiation exposure significantly alter the protection efficiencies. This study shows that the prevention of oxidative degradation is related to the susceptibility of antioxidant molecules for the inactivation of free radicals.

These results can be considered as a reliable test for different degradation conditions. The extension of our conclusive statements oner thermal degradation, photooxidation or radiochemical oxidation may be taken into consideration, and even differences between the concentrations of oxidizable intermediated exist for these ageing modes of packaging materials.

## Figures and Tables

**Table 1:** Melting temperatures (*T*_m_), temperatures at 5% mass loss (*T*_5%_) and residue % at 700 °C determined on EPDM composites.

Additive	*T*_m_ (°C)	*T_5%_* (°C)	Residue (%)
*Irradiation dose: 0 kGy*			
free	33.3	314.7	0
rosemary	34.3	370.7	0
POSS 1%	32.8	350.8	0.54
POSS 3%	33.4	268.7	1.10
POSS 5%	32.1	272.5	15.7
*Irradiation dose: 50 kGy*			
free	34.9	339.5	0
rosemary	34.3	350.6	0
POSS 1%	34.4	345.7	0
POSS 3%	33.7	285.3	1.18
POSS 5%	33.4	296.8	12.6

**Table 2:** The values of activation energies for the thermal stabilization of EPDM based on the values of oxidation induction times.

Additive	Testing Temperature (°C)	Oxidation Induction Time (min)	Relationship	Correlation Factor	Activation Energy (kJ mol^−1^)
free	160	42	y = −89.7 + 16.3x	0.99729	135.5
	170	20			
	180	8			
rosemary	170	502	y = −64.9 + 32.6x	0.99961	271.0
	180	98			
	190	18			
POSS 1 phr	160	254	y = −55.9 + 28.4x	0.99888	236.1
	170	65			
	180	14			
POSS 3 phr	160	25	y = −40.6 + 20.8x	0.99584	172.9
	170	10			
	180	3			
quercetin	170	710	y = −47.2 + 25.7x	0.99602	213.7
	180	240			
	190	58			
capsaicin	180	385	y = −60.1 + 23.7x	0.99614	197.0
	190	110			
	200	42			
oleanolic acid	160	50	y = −40.1 + 20.6x	0.99788	171.3
	170	15			
	180	6			

## Data Availability

Publicly available datasets were analyzed in this study. This data can be found in the cited references.

## Sample Availability

Samples of the compounds are not available from the authors.
